# Fabrication of Novel Printable Electrically Conductive Adhesives (ECAs) with Excellent Conductivity and Stability Enhanced by the Addition of Polyaniline Nanoparticles

**DOI:** 10.3390/nano9070960

**Published:** 2019-07-01

**Authors:** Jiayue Wen, Yanhong Tian, Chunjin Hang, Zhen Zheng, He Zhang, Zhipeng Mei, Xuanyi Hu, Yanqing Tian

**Affiliations:** 1 State Key Laboratory of Advanced Welding and Joining, Harbin Institute of Technology, Harbin 150001, China; 2 Department of Materials Science and Engineering, Southern University of Science and Technology, No. 1088, Xueyuan Road, Xili, Nanshan District, Shenzhen 518055, China

**Keywords:** Electrically conductive adhesive, polyaniline, electrical property and reliability, low-temperature bonding, printable circuits

## Abstract

Electrically conductive adhesives (ECAs) are one of the low temperature bonding materials. It can be used to replace toxic Sn-Pb solder. The key issue for the application of ECAs is how to improve their electrical properties. In the present study, we develop an effective method to promote the electrical properties of ECAs by addition of polyaniline (PANI) nanoparticles. PANIs were synthesized via a facile one-step chemical oxidative polymerization method. After adding 0.5 wt% PANI nanoparticles, the conductivity of ECAs increased dramatically by an order of magnitude. The bulk resistivity of 8.8 × 10^−5^ Ω·cm is achieved for 65 wt% silver fillers with 0.5 wt% PANIs loaded ECAs. Besides, this improvement has no negative effect on the shear strength and the aging life of ECAs. Moreover, the use of PANIs not only lowers the percolation threshold of ECAs, but also reduces the cost and improves the bonding reliability. Finally, PANIs enhanced ECAs patterns were successfully printed by a stencil printing method, which proved their potential applications in replacing conventional solder pastes and printing functional circuits.

## 1. Introduction

In order to avoid the hazardous influence of traditional Sn-Pb solder on human health, electrically conductive adhesives (ECAs), composed of polymer matrices and conductive fillers, are used as environmentally friendly substitutes during the bonding process [1,2]. Besides, ECAs show wider applications in low-temperature interconnection and fine-pitch packaging, and more compatibility with non-wetting substrates or flexible printed circuits (FPCs) [3,4]. Thus, ECAs are also widely applied in printable electronics [5], electromagnetic interference shielding materials [6], radio-frequency identification devices [7], thermal management materials [8], and so on.

However, an acknowledged shortcoming of ECAs is their higher electrical resistivity compared to conventional solders, which is usually considered to be caused by surface status (such as remaining organic lubricants), and the higher melting point of conducive fillers [9] compared with tin-based solders. Therefore, surface treatment technology is widely used to improve conductivity by reducing the metal oxide or decreasing the thickness of lubricant layers [10,11]. For example, halogen elements were used to remove the Ag_2_O in silver-based ECAs [12] and high-temperature treatment method was used to remove the organic layers on the surface of conductive fillers [13]. But bare silver fillers without an oxide layer may oxidize again during storage or thermal curing. The lack of lubricants would also cause higher viscosity and degraded fluidity.

Therefore, adding other materials, especially nanomaterials, became another strategy to improve conductivity [14,15,16]. From 0D nanomaterials (silver nanoparticles [17]) to 1D nanomaterials (silver nanowires [18] or CNTs [19]), from 2D graphene [20] to 3D structural nanomaterials (silver dendrites [21]), from conductors to insulators [22,23], mixing an appropriate second phase materials into ECAs can significantly improve conductivity. However, there are still many barriers in the application such as expensive synthesis costs of silver nanowires or poor dispersion of graphene.

Although improving the conductivity of ECAs is a core issue, the deterioration of fluidity [13], the decrease of shear strength [24], and the poorer thermal stability due to the addition of silver nanomaterials or graphene described in the previous paragraph should not be ignored. Consequently, enhancing the conductivity of ECAs without affecting other properties, such as reliability or printability, is also required for them to replace the traditional solders.

In addition, polyaniline (PANI) is one of the most promising intrinsic conducting polymers. Its conductivity can be as high as conductors by choosing appropriate dopants [25] and improving crystallinity [26]. Therefore, PANI composites showed growing potential in such applications as thermoelectric materials [27], conductive materials [28], supercapacitors materials [29], and electromagnetic interference shielding materials [30]. Besides, PANI could also serve as the coupling agent to improve the thermal, mechanical, and flame retardant properties of epoxy resin [31,32,33,34]. However, epoxy resin is the most-common adhesive used in ECAs due to its excellent bonding property, good environmental resistance, and excellent thermal properties [12,13]. Therefore, PANI is very likely to become a reinforcing material for ECAs. However, there is a lack of research on trace amount of PANI as a dopant to improve the electrical properties of silver-epoxy-based conductive composites.

Herein, we proposed a simple and effective method for improving the electrical conductivity of conventional ECAs by adding a small amount of PANI nanoparticles, namely PANIs-enhanced ECAs (PECAs). The PECAs hybrid composites, as a substitution of Sn-Pb solders, Pb-free solders, or conventional ECAs, possess relatively low bonding temperature and high reliability whilst lower cost. After adding a small amount of PANI nanoparticles, the conductivity of ECAs increased dramatically by an order of magnitude. Mechanical shearing tests and aging experiments were also firstly performed systematically to ensure stable properties of ECAs after enhanced by PANIs. Finally, we successfully printed advanced functional circuits with PECAs materials.

## 2. Materials and Methods

### 2.1. Materials

Aniline (ANI), sodium dodecyl sulfate (SDS), methylhexahydrophthalic anhydride (MeHHPA), 2,4,6-tris(dimethylaminomethyl) phenol (DMP-30) and acetone were purchased from Aladdin, Shanghai, China. Ferric chloride hexahydrate (FeCl_3_·6H_2_O) was purchased from Tianjin Fuchen Chemical Reagents Factory, Tianjin, China. Ag flakes with average diameters of 8–10 μm were purchased from Bolong Silver Industry, Changsha, China. Liquid bisphenol-A epoxy resin (E51) was purchased from Beijing Tonglian Hengxing Technology Co., Ltd., Beijing, China.

### 2.2. Synthesis of PANIs Nanoparticles

The PANI nanoparticles were synthesized by the chemical oxidation polymerization process and the size of the particles was adjusted by the usage of surfactants [35,36,37]. Typically, 1 mmol ANI and 10 mmol (SDS) were dissolved in 20 mL DI water. Then, 5 mL FeCl_3_·6H_2_O (1M) was added into the above solution. The solutions were stirred magnetically for 4 h while the vessel containing the solution was cooled in an ice bath. Finally, black emerald green precipitates were produced and washed several times with DI water and acetone. Then, the products, namely PANIs, were dried in a vacuum oven.

### 2.3. Preparation of ECAs

**Scheme 1:**
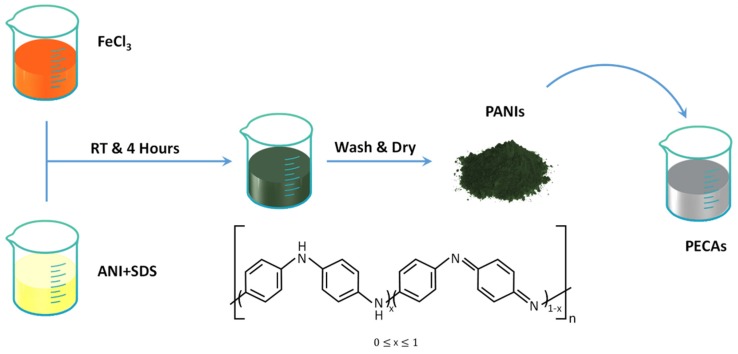
Illustration of the synthesis of polyanilines (PANIs) by chemical oxidation polymerization process and fabrication of PANIs-enhanced electrically conductive adhesives (PECAs) with excellent electrical property.

Epoxy was chosen as the resin matrix. Typically, the epoxy resin, MeHHPA (curing agent) and DMP-30 (curing catalyst) were mixed with a mass ratio of 100:86:0.5. Different amount of silver flakes were fixed into epoxy uniformly by a mixing deaerator (SIE-MIX80, Sienox Industrial Products Ltd. Guangzhou, China) at 2500 rpm for 5 min. Combined with our previous research [38], the curing temperature of the resin was set to 150 °C, which is very suitable for low temperature interconnect requirements on the field of electronic packaging. Simultaneously, different amount of PANIs (from 0 to 1.5 wt%) were also fixed into epoxy to enhance the performance of ECAs. Scheme 1 illustrated the integrated fabricating procedure of PANIs enhanced conductive adhesives composites. The films of ECAs on the surfaces of glass slides were prepared by knife coating for the resistivity test. High-conductivity tracks were stencil printed on the PET substrates with the thickness of 100 μm.

### 2.4. Characterization

Scanning electron microscopy (Helios Nanolab 600i, FEI NanoPorts, FEI Corporation, OR, USA) was used to characterize the morphologies of PANI nanoparticles operated at an accelerating voltage of 20 kV and an accelerating current of 2 μA. Energy dispersive X-ray spectroscopy (EDX, Helios Nanolab 600i, FEI NanoPorts, FEI Corporation, OR, USA) was used to analyze the elemental distributions of the composite. XRD patterns were collected by X-ray diffractometer (D8-ADVANCE, Bruker Corp., Rheinstetten, Germany) with Cu Kα radiation (λ = 1.5418 Å) at 40 kV and 40 mA. A four-point probes system (RTS-9, 4 PROBES TECH, Guangzhou, China) was used to test the sheet resistivity of ECAs films and then deducing electrical resistivity. For each condition, each sample was tested 10 times to calculate the average parameters and errors. Shear strength test was performed at room temperature by using a pull tester (DAGE 4000). The shear height of the blade tip above the Cu substrate was 0.5 mm and the shear speed was 100 μm/s.

## 3. Results and Discussion

### 3.1. Experimental Results and Discussion

**Figure 1:**
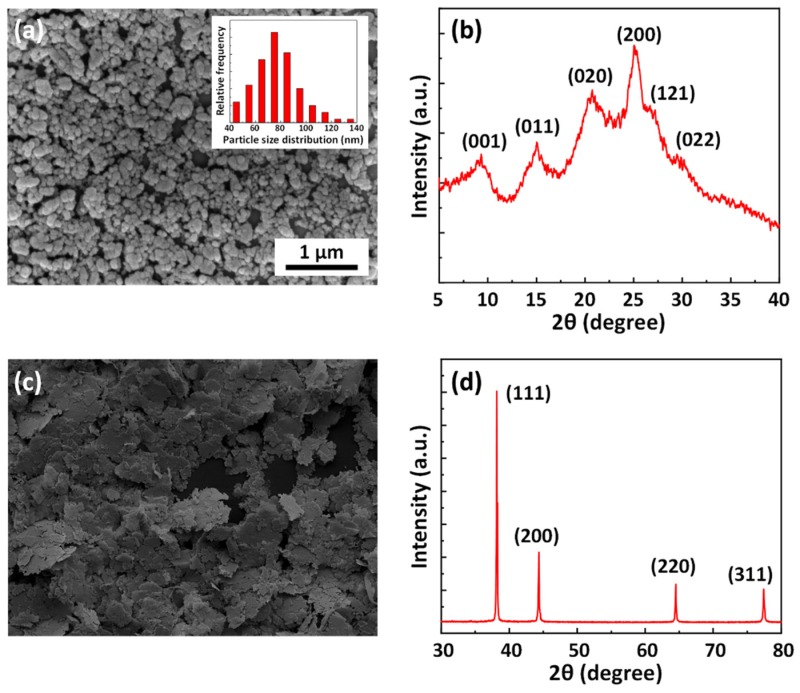
(**a**) SEM image of as-prepared nano-structure PANIs (insert: diameter distribution of PANI nanoparticles); (**b**) XRD spectra of as-prepared PANI materials; SEM image (**c**) and XRD spectra (**d**) of silver flakes.

When using suitable polymeric stabilizers, PANI particles can be protected from macroscopic aggregation and aniline would be polymerized into the spherical nanoparticles spontaneously [39]. Figure 1a shows the morphology of PANI nanoparticles and histograms of diameter distribution. The diameter of PANI nanoparticles was controlled to 77.0 ± 17.7 nm by dispersion polymerization. In the PANI X-Ray Diffraction specimen (Figure 1b), the crystalline peaks appeared at 2 θ = 9.1°, 14.8°, 20.6°, 25.1°, 26.5°, and 29.8° corresponding to (001), (011), (020), (200), (121), and (022) reflections [40]. As shown in Figure 1c, silver flakes with a diameter of 8–10 μm were selected as conductive fillers for conventional ECAs in this work. Figure 1d shows the XRD pattern of silver micro-flakes and the 2 θ peaks observed at 38.1°, 44.4°, 64.5°, and 77.4° corresponds to (111), (200), (220), and (311) reflection planes of a fcc lattice of silver.

### 3.2. Electrical Properties of PECAs

**Figure 2:**
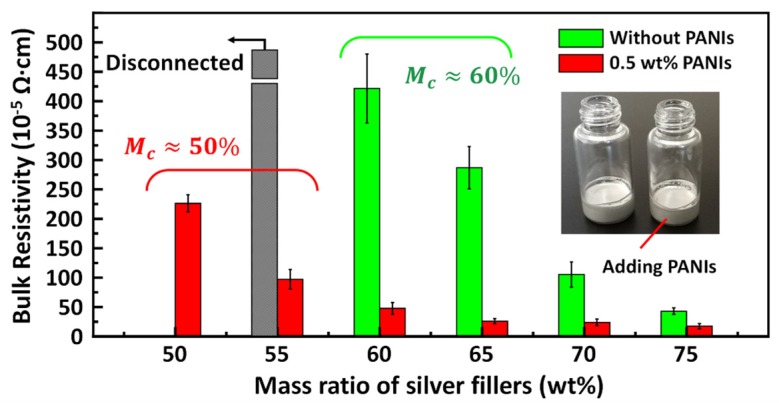
The comparison of bulk resistivity of electrically conductive adhesives (ECAs) between with 0.5 wt% or without PANIs dopants. Illustration shows the as-prepared silver-based ECA pastes with (**right**) or without (**left**) PANIs.

PECAs were prepared by adding a small amount of PANIs into conventional ECAs. As shown in the inset images of Figure 2, PECAs and conventional ECAs were similar in appearance. Figure 2 showed the comparison of conductivity of ECAs with and without 0.5 wt% PANIs dopants. Although the conductivity of ECAs also improves as more silver filler is added, resistivity of ECAs is further reduced by the addition of 0.5 wt% PANIs. The bulk resistivity decreased from 286.8 × 10^−5^ Ω·cm to 26.1 × 10^−5^ Ω·cm for 65 wt% silver-filled ECAs.

Macroscopically, the electrical resistance of ECAs conforms to percolation theory. The electrical conductivity of conductive composites follows the power–law relationship:

$$\sigma =\sigma_{0}{\left(M-M_{c}\right)}^{t}$$

where σ is the conductivity of the composite, $\sigma_{0}$ is the intrinsic conductivity of the filler, M is the mass fraction of the filler, $M_{c}$ is the critical percolation threshold, and t is the power law exponent [41]. The addition of PANIs could also be considered as reducing the percolation threshold of silver-flake-filled ECAs from about 60 wt% to lower than 50 wt%, which facilitates printing the pastes and reducing the cost of the ECAs.

**Figure 3:**
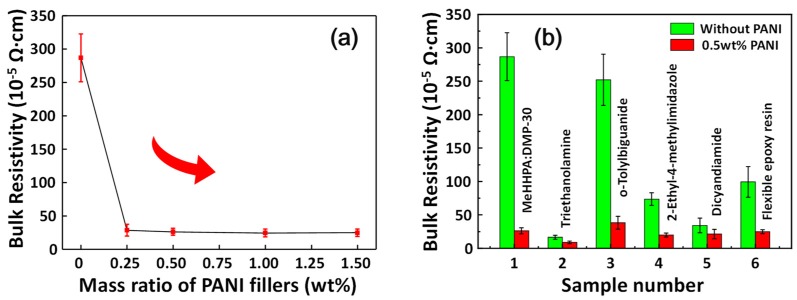
(**a**) The change trend of ECAs with adding different amount of PANIs; (**b**) Comparison of PANIs’ improvement effect on ECAs cured with different curing agents.

Figure 3a shows the change in ECAs resistivity (with 65 wt% silver fillers) as the mass ratio of PANI fillers is increased. A sharp resistivity drop (from 286.8 × 10^−5^ Ω·cm to 28.5 × 10^−5^ Ω·cm) occurred after as less as 0.25 wt% of PANIs were added. Further increasing PANIs to 1.5 wt%, the resistivity of ECAs remained stable. Another comparison in electrical resistivity of epoxy-based ECAs (with different curing agents) with and without 0.5 wt% PANIs was shown in Figure 3b. The method of adding PANIs to decrease the bulk resistivity of ECAs was suitable for various epoxy-based ECAs. The composition of each conductive pastes was listed in the Table 1. The highest conductivity could reach 8.8 × 10^−5^ Ω·cm.

For the resistance of conductive composites, there are three separate contributions to the resistance: constriction resistance at the contacts, tunneling resistance at the contacts, and the intrinsic filler resistance through each particle [42]. Tunneling resistance generally dominates the magnitude of the overall resistance:

$$R_{c}=R_{cr}+R_{t}=\rho_{i}/d+\rho_{t}/a$$

where $R_{c}$ is the composite resistance, $R_{cr}$ is the constriction resistance, $R_{t}$ is the tunneling resistance, $\rho_{i}$ is the intrinsic filler resistivity, d is the diameter of the contact spot, $\rho_{t}$ is the tunneling resistivity and a is the contact spot area.

For the conduction mechanism, the addition of conductive PANIs nanomaterials is equivalent to providing more contact points for conductive composites. In our previous research [38,43], we found that there was a strong interaction between PANIs and silver flakes, which could alter the distribution of the silver flakes in the resins and enhance the tunneling state between them.

### 3.3. Mechanical Property and Reliability

**Figure 4:**
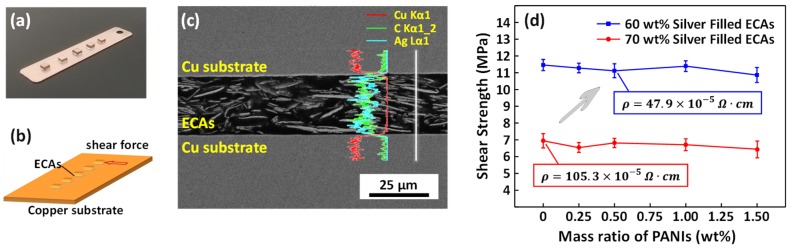
Photograph (**a**) and schematic diagram (**b**) of Cu/ECAs or PECAs/Cu sandwich structure for shearing test; (**c**) The cross section of Cu/PECAs/Cu bonding joint and EDS line scan corresponding to the write line; (**d**) dependence of the shear strength of ECAs joint on the amount of adding PANIs.

The shear strength of the ECAs after addition of PANIs was measured by mechanical test equipment. Some dummy dies adhered to the bare copper substrate and the Cu/ECAs or PECAs/Cu sandwich structures were formed to test the shear strength as shown in Figure 4a,b. Figure 4c shows the cross-section SEM view of the joints. The line scan Cu/Ag/C element distribution curves were also marked in Figure 4c. The conductive fillers uniformly dispersed in the resin and contacted each other to form conductive paths. Epoxy and the substrate formed a tightly bonded interface ensuring electrical and mechanical properties during the ECAs connection process. Figure 4d showed the shear strength changes of the ECAs with the various PANIs addition (the weight loading of silver micro-flakes was set at 60 wt% and 70 wt% respectively). In the case of 60 wt% silver and 0.5 wt% PANIs filled ECAs as an example, shear strength could reach 11.1 ± 0.4 MPa. First, the addition of PANIs did not have a negative impact on the shear strength of ECAs. Simultaneously, it was obvious that the more the resin matrix contained, the higher the bonding strength of the conductive adhesive was. Therefore, the shear strength could only reach 7 MPa for conventional ECAs with 70 wt% silver fillers with the bulk resistivity of merely 105.3 × 10^−5^ Ω·cm. In other words, PECAs had better bonding reliability when meeting the same conductivity requirements.

**Figure 5:**
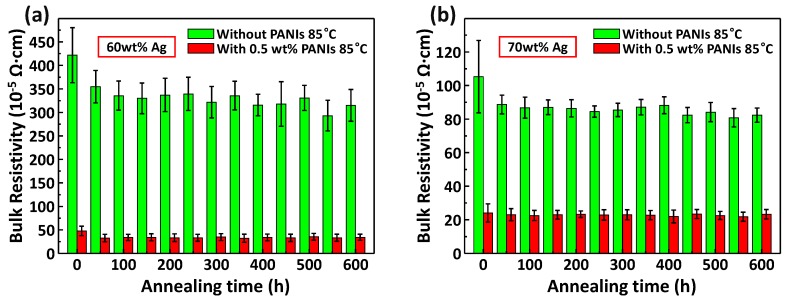
Bulk resistivity evolution of (**a**) 60 wt% and (**b**) 70 wt% silver-filled ECAs with or without 0.5 wt% PANIs during a 600-h aging test.

It was generally acknowledged that the performance of conductive adhesives in aging tests was not as good as solder bonding. Therefore, in order to ensure the reliability of PANIs enhanced ECAs, a 600-h aging experiment at 85 °C was performed and the electrical resistivity is shown in Figure 5a,b. The trend of electrical resistivity of ECAs with less silver fillers (60 wt%) and more silver fillers (70 wt%). For 70 wt% silver-filled ECAs with 0.5 wt% PANIs dopants, the final resistivity was stable at 23.3 × 10^−5^ Ω·cm compared to the original resistivity (24.1 × 10^−5^ Ω·cm). It was suggested that the electrical properties of PANIs enhanced ECAs would remain stable during long time high-temperature working process for the first time.

### 3.4. Applications

**Figure 6:**
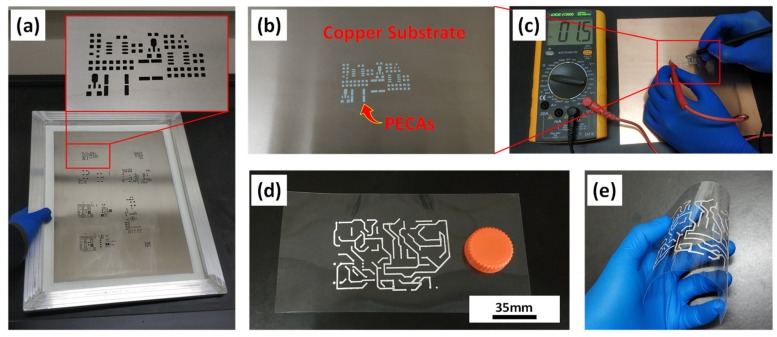
(**a**) Stencil printing mask prepared by laser beam cutting with the thickness of 100 μm; (**b**) Stencil printing PECA patterns corresponding to the mask on the copper substrate playing the same role as Sn-Pb solders pastes; (**c**) Photographs of the relative resistance of the PECAs after curing; (**d**,**e**) Photographs of printable flexible circuits on a PET film. The printed circuits still maintained excellent adhesion performance during the bending process.

For replacement of solder pastes, as shown in Figure 6a,b, fine-pitch PECAs pattern can be made by a stencil printing method on copper substrates. As a kind of interconnecting material instead of solders, PECAs have advantages of low process temperature and low environmental pollution. Figure 6c shows its excellent electrical conductivity after curing. Besides application as a kind of adhesive, advanced ECAs also need to meet the requirement in printable electronics and flexible electronics. Therefore, the adaptability of PANIs enhanced ECAs in printed electronic technologies was tested shown as Figure 6d,e. Since the viscosity of the conductive paste can be adjusted very easily, a radio circuit with fine-pitch and excellent conductivity can be easily printed on a flexible PET substrate.

## 4. Conclusions

We reported an innovative method of adding PANIs into epoxy-based ECAs for improving their electrical properties. PANIs, as a kind of intrinsic conducting polymers, were added into the ECAs in the state of nanoparticles for the first time to improve the electrical performance. The bulk resistivity can decrease to as low as 8.8 × 10^−5^ Ω·cm and the shear strength maintain 11.1 MPa, when incorporating 0.5 wt% PANIs into 65 wt% silver-filled ECAs. Moreover, the bulk resistivity of PANIs enhanced ECAs was stable in the 85 °C aging test. Therefore, PECAs showed better potential as a substitution for Sn-Pb solder with many advantages, such as low bonding temperature or fine-pitch, compared with traditional epoxy-based ECAs. Besides, flexible PECAs have extended application for printing circuits toward the more efficient and more environmentally friendly electronic field.

## Figures and Tables

**Table 1:** Comparison of PANIs’ improvement effect on ECAs cured with different curing agents.

No.	Resin Composition (Mass Ratio)	Curing Process	Resistivity Change (×10^−5^ Ω·cm)	
			Without PANIs	With 0.5 wt% PANIs
1	Epoxy: MeHHPA:DMP-30 = (100:85:0.5)	150 °C 1 h	286.8	26.1
2	Epoxy:Triethanolamine = (100:16)	120 °C 3 h	16.7	8.8
3	Epoxy:o-Tolylbiguanide = (100:14)	140 °C 2 h	252.2	38.3
4	Epoxy:2-Ethyl-4-methylimidazole = (100:3)	120 °C 1 h	73.6	19.8
5	Epoxy: Dicyandiamide = (100:8)	180 °C 1 h	34.1	21.4
6	Self-made modified flexible epoxy resin	RT	99.4	25.0
